# Profiling cytokines in peritoneal effluent through a targeted multiplex cytokine panel provides novel insight into the localized proinflammatory processes in patients undergoing peritoneal dialysis

**DOI:** 10.3389/fmed.2024.1463391

**Published:** 2024-10-09

**Authors:** Patrycja Okulewicz, Bartosz Wojciuk, Iwona Wojciechowska-Koszko, Leszek Domański, Edyta Gołembiewska

**Affiliations:** ^1^Department of Nephrology, Transplantology, and Internal Medicine, Pomeranian Medical University, Szczecin, Poland; ^2^Department of Immunological Diagnostics, Pomeranian Medical University, Szczecin, Poland

**Keywords:** chronic kidney disease, peritoneal dialysis, inflammation, IL6, effluent, proteomics

## Abstract

**Objectives:**

The number of relevant markers indicating local intraperitoneal inflammation in patients undergoing peritoneal dialysis (PD) is limited. Therefore, this study aimed to evaluate the compatibility of peritoneal effluent (PE) for proteomic analysis and assess its potential utility in immunoprofiling studies.

**Methods:**

This pilot study included six PD patients from the Peritoneal Dialysis Center, Department of Nephrology, Transplantology, and Internal Medicine in Szczecin, Poland. All patients were clinically stable, with no signs of infections or malignancy at the time of study. PE samples were collected during routine surveillance visits at the Peritoneal Dialysis Center. Proteomic analysis of the samples was conducted using the Olink^®^ (Olink Proteomics AB, Uppsala, Sweden) Target 48 Cytokine panel.

**Results:**

PE samples were successfully analyzed, with 28 out of 45 proteins found within the limit of quantitation (LOQ) and 32 out of 45 proteins detected above the limit of detection (LOD). No significant interference from the matrix was observed in the assay. Biomarkers associated with low-grade inflammation showed varied levels, and the observed patterns were comparable across all patients.

**Conclusion:**

This study suggests that utilizing a cytokine panel with relative quantification is a promising method for PE immunoprofiling.

## 1 Introduction

Systemic inflammation is widely known as a predictor of increased mortality in chronic kidney disease (CKD) patients, primarily due to cardiovascular events (1–3). Persistent inflammatory states in CKD result from several factors, including increased production and decreased clearance of uremic toxins and pro-inflammatory cytokines, oxidative stress, acidosis, recurrent infections (often associated with dialysis access), malnutrition, and intestinal dysbiosis (4). As the estimated glomerular filtration rate (eGFR) declines below 15 mL/min per 1.73 m^2^ or with the onset of uremic symptoms—termed end-stage renal disease (ESRD)—kidney replacement therapy (KRT) becomes necessary.

In peritoneal dialysis (PD), a specific hyperosmotic solution is introduced into the peritoneal cavity, allowing uremic toxins and excess fluids to diffuse across the patient’s peritoneum. In addition to systemic inflammation, the topic of local, low-grade inflammation in PD patients is increasingly attracting attention (3, 5).

Chronic intraperitoneal inflammation is a consequence of multiple factors, such as bowel edema, bacterial translocation from overhydration, endotoxin accumulation, dietary and pharmacological challenges, genetic and epigenetic influences, and bioincompatibility of dialysis fluid. This ongoing inflammation may contribute to changes in peritoneal membrane transport, promote fibrosis, and ultimately reduce the efficacy and longevity of PD (6, 7).

Growing evidence links several biomarkers—C-reactive protein (CRP), interleukin 6 (IL6), tumor necrosis factor *α* (TNFα), interleukin 1 (IL1), and matrix metallopeptidase 12 (MMP12)—to low-grade inflammation (6–9). However, only a limited number of these biomarkers have been measured intraperitoneally, with IL6 widely recognized as an independent intraperitoneal inflammatory marker (5, 10).

Few exploratory studies have investigated the proteome of peritoneal fluid in conditions such as endometriosis, ovarian neoplasms, or abdominal sepsis (11–13). However, there is scarce research on the proteome of peritoneal effluent (PE) in patients undergoing PD, and the impact it exerts on patient and technique survival. Moreover he reliable methods for analyzing PE are lacking.

Targeted proteomic assays for various body fluids remain limited in scope. The Olink Target 48 Cytokine Panel is a high-multiplex, rapid-throughput biomarker analysis platform designed to detect 45 proteins associated with inflammation-related diseases in various sample types, such as serum or urine. This technology has been thoroughly validated, covering several key criteria, including assay precision (measured by the coefficient of variation, CV), the analytical range defined by a 32-point standard curve, and the establishment of limits such as the limit of detection (LOD), lower limit of quantification (LLOQ), and upper limit of quantification (ULOQ). Additionally, the platform has been tested for specificity and scalability. In this study, we aimed to evaluate the use of peritoneal effluent as a novel sample type for proteomic analysis, specifically to assess its potential utility in immunoprofiling with a focus on biomarkers of low-grade inflammation.

## 2 Materials and methods

### 2.1 Demographic data

Six patients with end-stage renal disease (ESRD) undergoing PD at the Peritoneal Dialysis Center, Department of Nephrology, Transplantology, and Internal Medicine in Szczecin, Poland, were enrolled in this study. Five patients were on continuous ambulatory peritoneal dialysis (CAPD), with four fluid exchanges daily using 2 L of 1.36% glucose-based dialysate containing 1.25 mmol/L of calcium (Baxter Healthcare or Fresenius Medical Care).

One patient was on continuous cyclic peritoneal dialysis (CCPD), using two 5 L bags of 1.36% glucose-based dialysate with 1.25 mmol/L calcium, along with a 2 L last bag (Baxter Healthcare). All participants were clinically stable with no signs or symptoms of overt infection or malignancy. None of the patients presented the symptoms of peritonitis at the time of the study or in the 4 weeks preceding it. Written informed consent was obtained from all participants.

The patients’ charts were reviewed to collect the following parameters: age, gender, weight (measured after draining dialysate), body mass index (BMI), type of nephropathy, presence of diabetes, residual renal function, markers of renal function (blood urea and creatinine levels), and dialysis vintage. Additionally, the results of peritoneal equilibration tests conducted within the preceding 6 months were recorded, along with any prior episodes of PD-related peritonitis.

The main characteristics of the study group, including the achieved IL6 concentrations in the PE, are presented in Table 1.

**Table 1:** Patient characteristics and corresponding Interleukin 6 concentrations in peritoneal effluent.

Patient	Gender	Age (years)	Cause of CKD	Type of PD	Dialysis vintage (months)	Serum creatinine concentration (mg/dL)	Past peritonitis	IL6 PE concentration (pg/mL)
1	Men	42	Congenital kidney anomalie	CCPD	8	10.73		28.0742
2	Men	76	DM2	CAPD	3	3.08		66.38757
3	Men	45	DM1	CAPD	42	10.39	x	6.37493
4	Men	49	amyloidosis	CAPD	18	5.83		9.42094
5	Men	65	IgA nephropathy	CAPD	8	7.49		13.42835
6	Women	78	DM2	CAPD	20	2.8	x	24.85172

DM1, diabetes mellitus type 1; DM2, diabetes mellitus type 2; CCPD, continuous cycling peritoneal dialysis; CAPD, continuous ambulatory peritoneal dialysis; x, recorded peritonitis.

### 2.2 Biochemical data

On the day of the study, 50 mL of PE was collected from the patients during the morning hours, after 6–8 h of its presence in the peritoneal cavity. The samples were immediately transferred to the laboratory, where they were vortexed and aliquoted into 1-ml tubes. These aliquots were then stored at −70°C until further analysis.

### 2.3 Proteomic analysis

Proteins were measured using the Olink^®^ Target 48 Cytokine Panel* (Olink Proteomics AB, Uppsala, Sweden), which employs Proximity Extension Assay (PEA) technology to simultaneously analyze 45 analytes with only 1 μL of each sample. In this method, pairs of oligonucleotide-labeled antibody probes bind to their respective target proteins. When the probes are brought in close proximity, the oligonucleotides hybridize in a pair-wise manner.

The addition of DNA polymerase triggers proximity-dependent DNA polymerization, generating a unique PCR target sequence. The resulting DNA sequence is subsequently detected and quantified using a microfluidic real-time PCR instrument (Biomark HD, Fluidigm, San Francisco, CA, USA).

The data are then subjected to quality control and normalization using internal extension controls and calibrators in order to adjust for intra- and inter-run variations. Each sample plate is evaluated based on the standard deviation of NPX (normalized protein expression, Olink’s arbitrary unit for relative protein quantification) values for incubation and detection controls. Only data from runs that meet these quality control criteria are reported. Moreover, in the sample control, each assay is analyzed for the accuracy of the calculated mean concentration. The final assay results are expressed in standard units (pg/ml), using a 4-parameter logistic (4-PL) fit for absolute quantification.

In addition to the validation procedures mentioned above, Olink’s T48 panel has an inbuilt QC system that allows users to monitor the technical performance of the assay and the quality of the samples themselves. The performance of this inbuilt control system was used to determine that there was no interference on the assay from the effluent. We checked for background effects from the matrix by conducting tests with blank perfusate samples, ensuring that the observed signal in the effluent was actually caused by signals surpassing the background in the matrix of interest.

We implemented heatmapping and radar plots to visualize the relationships between specific cytokine levels. Cytokines detected below the LOD, except for TNFα, were excluded from the heatmap analysis. Although the concentrations reported below the LOD were represented numerically, TNFα was not excluded due to its known impact on low-grade inflammation and to investigate its relationship with other cytokines. It is important to note that the LODs varied for each analyte.

## 3 Results

During the analysis of peritoneal dialysis effluents using the Olink^®^ Target 48 Cytokine panel, all samples and assays, except for Interferon ɣ (INF ɣ), passed the quality control (QC) criteria. Moreover, 62% of the proteins (28 out of 45) were detected within the LOQ, and 71% of the proteins (32 out of 45) proteins were detected above the LOD. The internal controls, spiked into every well of the plate, did not show observable interference.

The detectable cytokines (above LOD) were as follows: chemokine ligand 8 (CCL8), oxidized low-density lipoprotein receptor 1 (OLR1), chemokine ligand 9 (CXCL9), transforming growth factor alpha (TGFα), IL6, tumor necrosis factor ligand superfamily member 12 (TNF-SF12), eotaxin (CCL11), hepatocyte growth factor (HGF), Fms-related tyrosine kinase 3 ligand (FLT3LG), interleukin 7 (IL7), interleukin 18 (IL18), C-C motif chemokine 13 (CCL13), tumor necrosis factor ligand superfamily member 10 (TNFSF10), C-X-C motif chemokine 10 (CXCL10), INF ɣ, C-C motif chemokine 19 (CCL19), interleukin 15 (IL15), C-C motif chemokine 3 (CCL3), interleukin 8 (CXCL8), MMP12, granulocyte-macrophage colony-stimulating factor (CSF2), vascular endothelial growth factor A (VEGFA), interleukin 17C (IL17C), C-C motif chemokine 2 (CCL2), interleukin-17A (IL17A), oncostatin-M (OSM), macrophage colony-stimulating factor 1 (CSF1), C-C motif chemokine 4 (CCL4), C-X-C motif chemokine 11 (CXCL11), lymphotoxin-alpha (LTA), C-C motif chemokine 7 (CCL7), and interstitial collagenase (MMP1). The mean effluent IL6 concentration was 24.76 ± 22.12 pg./mL. Cytokines below LOD were as follows: interleukin 33 (IL33), stromal cell-derived factor 1 (CXCL12), interleukin 27 (IL27), interleukin 2 (IL2), interleukin-1 beta (IL1B), interleukin 4 (IL4), thymic stromal lymphopoietin (TSLP), interleukin 17F (IL17F), interleukin 13 (IL13), interleukin 10 (IL10), TNF *α*, granulocyte colony-stimulating factor (CSF3), and granulocyte colony-stimulating factor (EGF).

**Figure 1:**
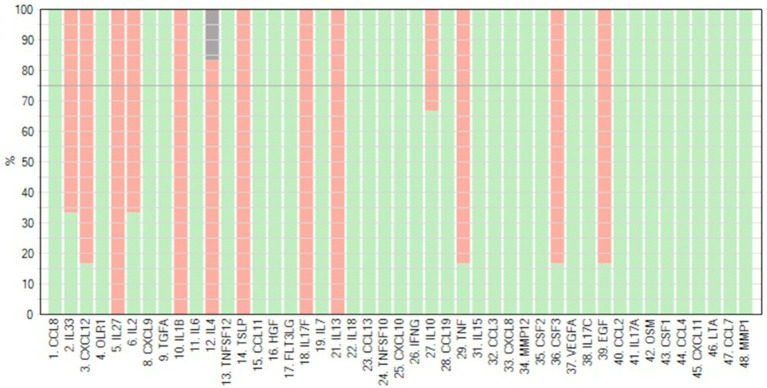
Detectability of 45 cytokines was measured using the Olink^®^ Target 48 panel in peritoneal effluent of PD patients. Red bars indicate the percentage detectability for each assay (generated by Olink^®^).

The percentage detectability of the examined proteins in patients’ PE is shown in Figure 1.

**Figure 2:**
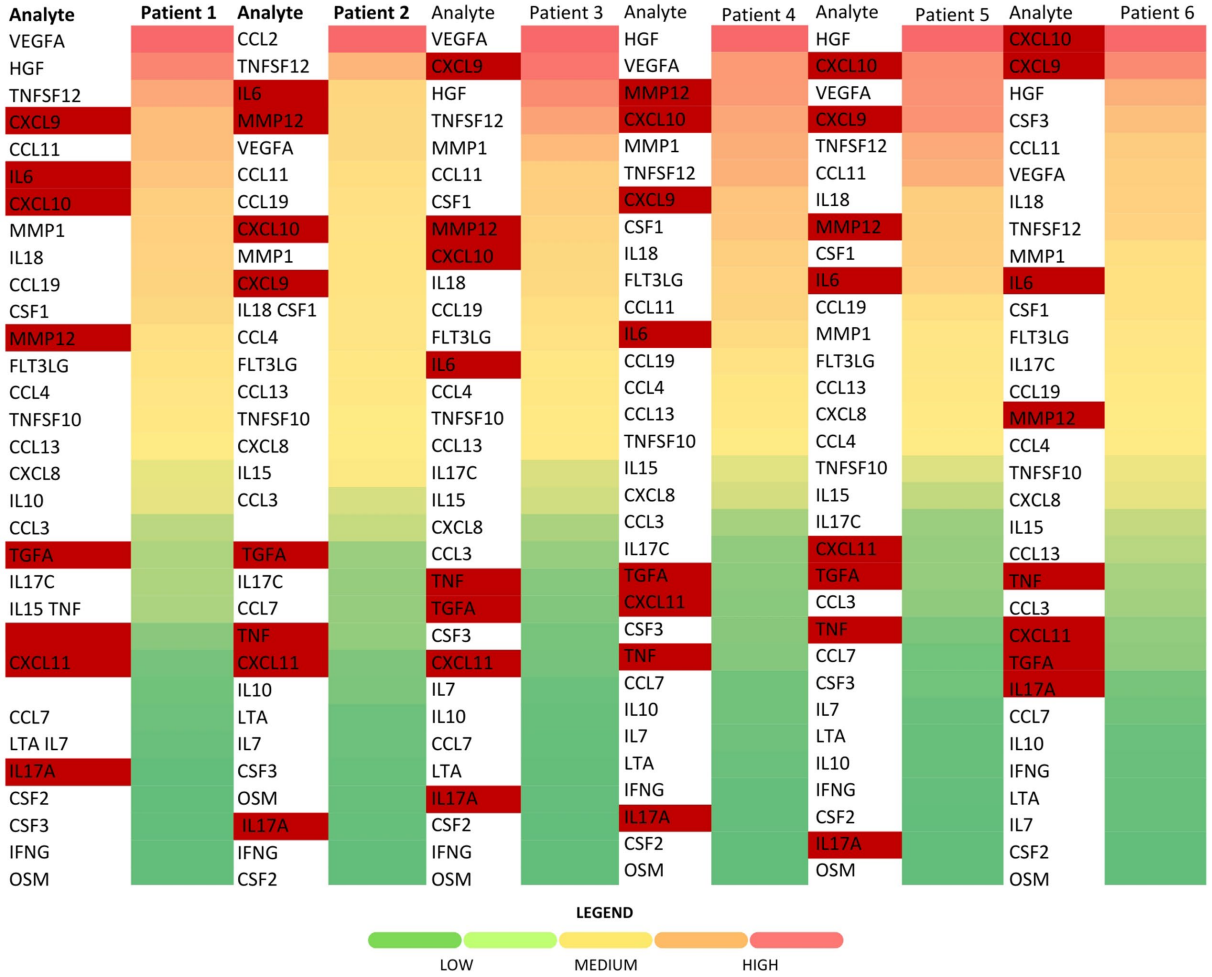
Heatmap of cytokine concentrations among the patients. Low-grade and chronic inflammation markers of interest are highlighted in red (visualized using Microsoft Excel).

The ranking of particular analytes across the samples was based on the heatmap and placed the majority of factors associated with low-grade inflammation—such as IL6, MMP12, CXCL10, and CXCL9—among the higher values. However, while CXCL11 and TGFα were detected above the LOD, they ranked near TNF, and similarly, IL17A was ranked below (Figure 2).

**Figure 3:**
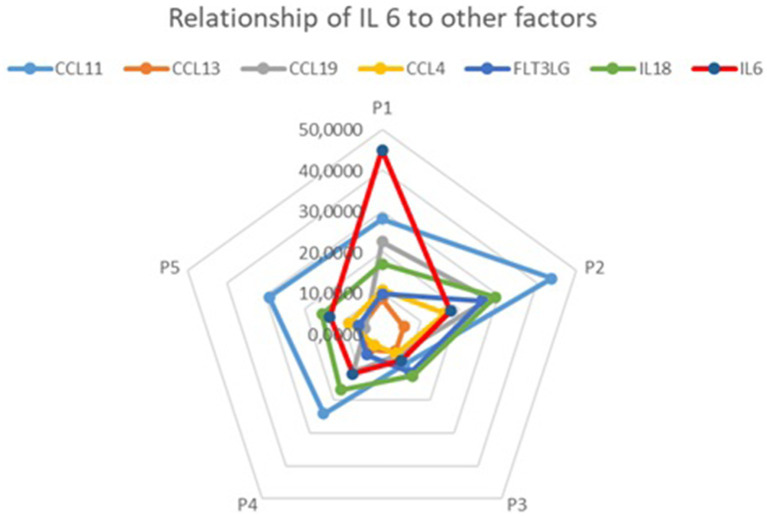
Relationships between IL6 and CCL11, CCL13, CCL19, CCL4, FLT3LG, and IL18 concentrations (visualized using Microsoft Excel).

In further analysis, we also investigated the relationship between IL6 and cytokines not traditionally recognized as markers of low-grade inflammation. We found that CCL11, CCL13, CCL19, CCL4, FLT3LG, and IL18 followed a similar pattern to IL6 in five out of the six analyzed samples, as shown in Figure 3.

## 4 Discussion

Peritoneal fluid, similar to urine and pleural effusion (14), holds special clinical significance in the context of inflammation, making its examination valuable for illustrating local inflammatory response in PD patients, particularly in relation to this type of KRT.

To the best of our knowledge, this pilot study is the first to demonstrate the detectability of 32 inflammatory cytokines in PE samples, establishing a foundation for using this matrix as a representative tool for investigating local inflammation in PD patients.

To date, research on markers relevant to intraperitoneal inflammation has primarily focused on specific cytokines and their impact on the peritoneal solute transport rate (PSTR), which has been further linked to patient survival (7, 15). However, Jelicic et al. (16) used measurements of IL6 and soluble IL6 receptor (sIL-6R) to examine whether local inflammation had a significant impact on daily diuresis and residual renal function in PD patients (16).

The study showed that IL6 levels were significantly higher in the peritoneal effluent (7.87 pg./mL) than in the serum (1.29 pg./mL), a finding later confirmed in the study of Fijałkowski et al. (17). Furthermore, Oh et al. (10) reported that IL6 effluent levels did not correlate with serum IL6 or CRP concentrations (10). In current times, IL6 is widely recognized as an independent marker of intraperitoneal inflammation (5). Our results (24.76 ± 22.12 pg./mL) are consistent with those reported by Jelicic et al. (16) (7.87 ± 2.62 pg./mL) and Fijałkowski et al. (17) (30.7 ± 24.6 pg./mL). This is particularly important as it demonstrates that the PEA-based quantification method is technically compatible with PE and can successfully quantify inflammatory cytokines. This supports further investigations of relationships between different cytokines within a sample and between samples.

Interestingly, in our study, concentrations of several other biomarkers (CCL11, CCL13, CCL19, CCL4, FLT3LG, and IL18) followed the same pattern as IL6 concentration on the radar chart. Although current reports on their associations with low-grade inflammation in patients with PD are lacking, it has been found that serum CCL11 levels are higher in CKD patients (18), potentially promoting interstitial inflammation in diabetic nephropathy and correlating with eGFR decline (19). Mangieri (20) study also demonstrated higher serum CCL11 levels in patients with idiopathic retroperitoneal fibrosis. Additionally, another mediator, IL18, was shown to increase both locally and systemically in rats subjected to PD (21). While these results should be interpreted cautiously, we hypothesize that further research with a larger study group may identify CCL11, CCL13, CCL19, CCL4, FLT3LG, and IL18 as potential predictors of low-grade inflammation.

Our analysis found that the dialysate concentrations of IL1β and TNF were below the LOD in the majority of samples, which is in line with the findings of Oh et al. (10). However, since LODs varied between different cytokines, the relatively high LOD for TNF should be taken into account when interpreting the heatmap rankings. This is particularly evident in relation to IL17.

Wang et al. (22) analyzed the peritoneal fluid of patients with PD-related peritonitis and, contrary to our findings, reported high effluent levels of IL17 in that patient group. They suggested that IL-17 can serve as an early marker of immune response (22). Similarly, Witowski et al. (23) demonstrated the impact of IL-17 on peritoneal mesothelial cells and peritoneal vascularity, highlighting its significant role in local inflammation and angiogenesis (23). It is important to note that the patients in our study did not experience peritonitis during the study or in the 4 weeks preceding it.

These findings support the hypothesis that analyzing peritoneal effluent plays a crucial role not only in understanding the dynamics of intraperitoneal inflammation but also in preventing its detrimental consequences. The use of precise and efficient proteomic techniques can contribute significantly to advancements in this field. In this study, we identified 28 proteins within the LOQ, revealing multiple biomarkers and patterns of unknown significance in PD patients. However, due to the small sample size, the quantitative results could not be statistically analyzed, which is a limitation of the study. Despite this limitation, the results are promising and encourage further research.

## 5 Conclusion

We successfully identified that the majority of targeted biomarkers in PE above the LOD, including several well-recognized markers of low-grade inflammation, such as IL6, were found in high concentrations. Additionally, the levels of certain biomarkers were observed to correlate with IL6. The present study suggests that PE immunoprofiling can be a useful method in the explorative analysis of intraperitoneal inflammatory processes in patients undergoing PD. This approach holds potential for both advancing our understanding of pathophysiological mechanisms and improving clinical care.

## Data Availability

The original contributions presented in the study are included in the article/supplementary material, further inquiries can be directed to the corresponding author.
